# Incidental finding of Cowper’s syringocele in traumatic blunt injury: a case report

**DOI:** 10.1093/jscr/rjae250

**Published:** 2024-04-26

**Authors:** Lindsey A Braden, Katherine Quibell, Amber L Jones

**Affiliations:** Division of Trauma and Acute Care Surgery, Department of Surgery, Kern Medical Center, 1700 Mount Vernon Avenue, Bakersfield, CA 93306, United States; Department of Surgery, Harbor-UCLA Medical Center, 1000 West Carson Street, Torrance, CA 90509, United States; Division of Trauma and Acute Care Surgery, Department of Surgery, Kern Medical Center, 1700 Mount Vernon Avenue, Bakersfield, CA 93306, United States; Osteopathic School of Health Medicine, Western University, 309 E 2nd St, Pamona, CA 91766, United States; Division of Trauma and Acute Care Surgery, Department of Surgery, Kern Medical Center, 1700 Mount Vernon Avenue, Bakersfield, CA 93306, United States

**Keywords:** bulbourethral gland dilation, urogenital sinus gland, LUTS

## Abstract

Cowper’s syringocele is a cystic dilation of the bulbourethral duct or gland. This rare pathology has historically been diagnosed in the pediatric population, with recent literature reporting an increased incidence in adults. Attempts have been made to classify Cowper’s syringoceles by their appearance on imaging and endoscopy, however a simpler classification of unobstructed versus obstructed may be of more utility in directing management. Herein, we present a novel case of a Cowper’s syringocele developed in adulthood that supports the suspicion for underdiagnosis, as well as one proposed mechanism of acquired etiology of Cowper’s syringoceles involving incomplete or intermittent obstruction secondary to the application of external force of the leading to asymptomatic cystic dilation.

## Introduction

Cowper’s or Bulbourethral glands are paired, male accessory sexual organs positioned within the bulbospongial tissue at the level of the urogenital diaphragm and lie posterolateral to the membranous urethra. The glands coalesce to form ducts approximately 2.5 cm long, which merge to penetrate the corpus spongiosum and open midline into the ventral aspect of the bulbar urethra. These glands secrete an alkaline mucinous fluid that is released prior to ejaculation, serving to lubricate the urethra, coagulate semen, and neutralize the acidity of the vagina [1–3]. Although rare, a number of symptomatic and asymptomatic congenital and acquired pathologic diseases have been described [1].

Cowper’s syringoceles are an uncommon and likely underappreciated pathology involving the cystic dilation of the ducts or gland, which falls along a spectrum if severity. Historically, the disease has been described primarily in the pediatric population [4]. However, in recent years, there has been an increasing number of cases presented in literature involving adult development of these cysts. Herein, we report a rare case of an adult, asymptomatic Cowper’s syringocele incidentally discovered in 31-year-old man suffering from blunt trauma who was referred to the authors’ facility as a tier two trauma activation.

## Case presentation

**Figure 1:**
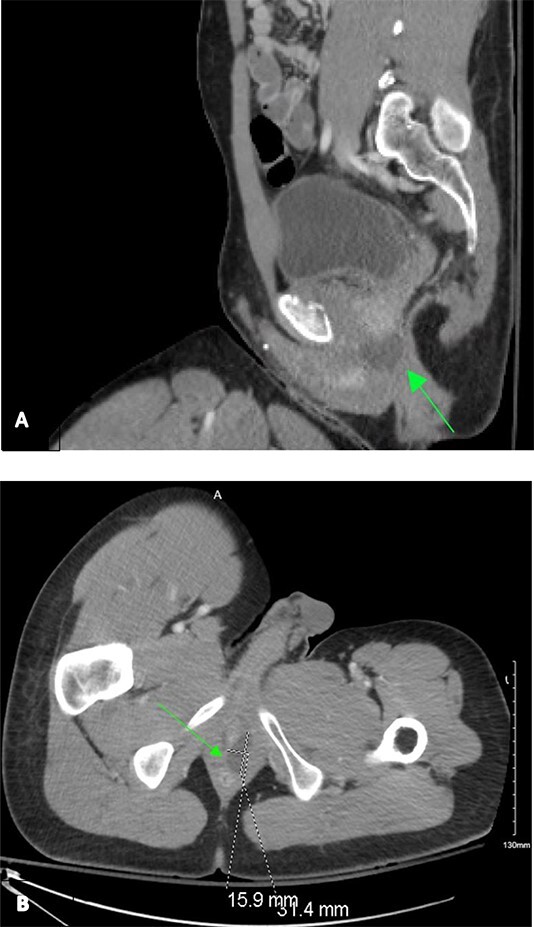
Contrast-enhanced CT chest/abdomen/pelvis during a tier 2 trauma activation, revealing a homogenous hypodense perianal fluid collection from sagittal (A) and transverse (B) planes.

A 31-year-old African American man with history of schizophrenia and prior traumatic amputations of the left upper and left lower extremities was referred to the Emergency Department as a tier 2 trauma activation following a blunt injury in an auto versus pedestrian collision, where he was a wheelchair-bound pedestrian hit by oncoming traffic. On presentation, the primary survey was unremarkable and noted to be hemodynamically stable. Extended Focused Assessment with Sonography in Trauma exam showed no free fluid. Secondary survey was remarkable for pelvic tenderness. He endorsed diffuse midline cervical, thoracic, and lumbar tenderness with palpation. However, no step-offs, deformities, or external signs concerning for traumatic spinal cord injury were noted. Given the mechanism of injury, as well as the secondary exam findings, computed tomography (CT) of the chest, abdomen and pelvis was obtained. Imaging revealed a non-operative left superior ramus fracture and a 31 mm by 16 mm left homogenous cystic lesion, situated at base of the prostate and spanning from the anorectal junction to the urogenital sinus (Fig. 1).

After the patient’s stability from a trauma standpoint was ensured, further investigation of the seemingly unrelated perianal fluid-filled collection ensued. Thorough collateral history was difficult to obtain given the patient’s psychiatric history. Patient history and chart documentation revealed no refractory pain, difficulty with urination or defecation, hematuria, dysuria, rectal or peri-rectal drainage, hematochezia, melena, fevers or chills. Imaging from a hospitalization three years prior, including a CT of the abdomen and pelvis, was reviewed and showed no homogenous cystic perianal mass.

Digital rectal exam and transrectal ultrasound were attempted, however, the patient was unable to tolerate lateral decubitus positioning in the setting of his known pubic ramus fracture. Due to the location and characteristics of the fluid collection, the case was discussed with colorectal surgery. Further investigation during the current hospitalization was felt to be warranted, as a perianal abscess could not be ruled out. Additionally, the patient had multiple identified impediments to accessing future care, including significant social factors and a prior history of loss to follow up. Recommendations were discussed with the patient, including ultrasonography, magnetic resonance imaging (MRI) and additional evaluation by urology for possible cystourethrogram. The patient possessed capacity, verbalized understanding of the treatment options and outcomes, but remained firm in his desire for prompt discharge upon arrival of the durable medical equipment. Due to patient preference, the decision was made to proceed to the operating room for exam under anesthesia with possible biopsy and drainage of the perianal abscess.

In the operating room, a rigid proctosigmoidoscope was inserted 20 cm and insufflated, and on slow withdrawal, the rectal and anal walls were found to be without defect, diverticula or other pathology. Given that the patient was taken promptly to the operating room, an intraoperative transrectal ultrasound transducer was not readily available at the authors’ facility. However, due to the fluid collection’s proximity abutting the anorectal wall, a safe window was readily accessible and thus the decision was made to obtain a fluid aspirate at that time. A 16-gauge needle was then inserted above the anal margin into the homogenous mass and approximately 4 cc of straw-color, mucinous material was aspirated and sent for further cytology studies, gram stain and culture analysis. The procedure was then terminated, and patient recovery was uneventful. Cytology of the aspirated fluid revealed mucoid, hypocellular fluid with lymphocytes and macrophage predominance without evidence of acute inflammation or malignancy.

Post-operatively, the patient insisted on discharge home directly following the procedure, from the post-anesthesia care unit. Given his stability, this was deemed appropriate with close follow up. Unfortunately, despite multiple attempts to contact him via phone and mail, the patient was ultimately lost to follow up. However, to date, the patient has returned to the authors’ facility for unrelated complaints one most following surgery. At that time, he declined any post-operative urogenital or rectal complaints and has remained asymptomatic.

## Discussion

Cowper’s syringoceles are thought to be either a congenital or acquired pathology, with earlier presentation in pediatric patients believed to be more likely due to underlying congenital anomalies, versus acquired in the adult population [4, 5]. However, the underlying mechanism of formation of each is not well understood.

An early classification system was outlined by Maizels et al. in 1983, based on radiologic and endoscopic appearance of the syringoceles, and included four categories: a simple syringocele with minimal ductal dilatation; a perforate syringocele with a bulbous duct draining into the urethra as an apparent diverticulum; an imperforate syringocele with a bulbous duct resembling a submucosal cyst and appearing as a radiolucent mass; and a ruptured syringocele with residual membrane remaining in the urethra following rupture of a dilated duct [1, 6, 7]. In recent years, a simpler classification system has been developed, dividing syringoceles into obstructive versus non-obstructive, and references presenting symptoms as well as configuration of the duct’s orifice compared to the urethra [4].

In normal embryologic development, endodermal epithelium in the urogenital sinus is induced by dihydrotestosterone to form buds that eventually become the Cowper’s ducts around week 11; by week 16, differentiation occurs into compound tuboalveolar secretory tissue [7]. In mice, insufficient transforming growth factor-beta 2 has been shown to interrupt this normal developmental pathway and lead to syringocele formation [2]. Other studies have shown a high incidence of Cowper’s syringocele associated with urethral stricture, particularly at the proximal and middle bulbar urethral junction [7, 8]. Additional studies have demonstrated an association between syringoceles and posterior urethral valves, which may be due to the overgrowth of epithelium leading to both pathologies [8]. Any of these mechanisms individually or in combination may reasonably contribute to syringocele formation, though depending on the degree of anomaly, may or may not result in a detectable pathologic state. Increasing availability of advanced imaging such as MRI has therefore revealed a higher prevalence of these syringoceles than previously thought, likely secondary to incomplete pathologies that do not necessarily cause symptoms, or cause symptoms to emerge later in life.

In the case of an acquired syringocele, etiologies may include isolated or superimposed trauma caused by prolonged urethral catheterization, post-inflammatory ductal changes after infection, and stasis pressure changes that lead to obstruction of bulbourethral glands into the urethra, mucous accumulation and cystic dilation [3, 4].

The disease most frequently presents in the pediatric population spanning from the neonatal period to late adolescence, with presenting symptoms correlated with the patient’s age; these include dysuria, urinary tract infection, hematuria, urethral discharge, urgency, post-void dribbling, poor urinary stream, urinary incontinence, scrotal abscess or scrotal swelling [6]. In adults, presenting complaints similarly include voiding dysfunction and perineal swelling. Between the two populations, symptoms at presentation differ and lie along a continuum of severity, with children generally presenting with more severe symptoms and adults presenting with more indolent courses.

There is no current consensus as to the best diagnostic or treatment approaches, though syringoceles are often first detected via ultrasound or CT. Ultrasonography followed by MRI is generally considered the preferred confirmatory imaging modality. Additionally, antegrade or retrograde cystourethroscopy is delineated as the gold standard for confirming diagnosis [2, 6, 7]. Symptomatic syringoceles are generally managed surgically, but often will undergo a trial of conservative management prior to intervention [2, 6].

In the described case, our patient declined the aforementioned diagnostic testing. Furthermore, although the patient was asymptomatic and lacked refractory pain, fluid analysis of the collection was deemed appropriate and promptly obtained in the setting of his complicated psychosocial factors and inability to rule out a perirectal abscess. Aspiration of the fluid yielded low suspicion for microbial pathology and reassured the suspected diagnosis of an asymptomatic Cowper’s syringocele. Thus, further interventions were not warranted, given the asymptotic nature as well as confirmatory findings on imaging and cytologic fluid analysis.

While our patient was lost to the follow-up and detailed medical history could not be ascertained in its entirety, a review of records from prior hospitalizations revealed multiple risk factors for development of a Cowper’s syringocele. Comparing the present CT images with those available from three years prior, interval development of the syringocele was confirmed. At that time, he sustained multiple traumatic injuries including a left below the knee amputation. Factors including the patient’s abrupt immobility, prolonged urethral catheterization during hospitalization, and wheelchair dependence provide support for one possible mechanism of Cowper syringocele formation in adults, whereby sustained external pressure causes progressive obstruction of the duct orifice and leads to cystic dilation of the duct.

The asymptomatic nature of the described case aligns most closely with an open syringocele lacking complete ductal occlusion, which could therefore drain, albeit incompletely, leading to ductal dilation. Aspiration alone of a syringocele does not comprise true definitive treatment as defined by the existing literature. However, in the presented case, the patient refused further intervention; given the asymptomatic nature, aspiration may prove sufficient in cases in which there is incomplete or intermittent ductal obstruction that does not lead to development of symptoms [7].

## Conclusion

Cowper’s syringocele is an uncommon and likely underdiagnosed cystic dilation of the Cowper’s duct that carries a spectrum of severity. Congenital abnormalities more commonly lead to symptomatic disease in early childhood, while acquired abnormalities may or may not manifest with symptoms that prompt the patient to seek medical treatment. The presented case supports the shifting suspicion that the rarity of Cowper’s syringoceles may perhaps be attributed in part to the disease’s underdiagnosis, as suggested by the increasing incidence in adults presented in recent literature.

## Data Availability

All data related to the content of this case report is present within the body of the manuscript.
